# Can Static Habitat Protection Encompass Critical Areas for Highly Mobile Marine Top Predators? Insights from Coastal East Africa

**DOI:** 10.1371/journal.pone.0133265

**Published:** 2015-07-17

**Authors:** Sergi Pérez-Jorge, Thalia Pereira, Chloe Corne, Zeno Wijtten, Mohamed Omar, Jillo Katello, Mark Kinyua, Daniel Oro, Maite Louzao

**Affiliations:** 1 Global Vision International Kenya (GVI) P.O. Box 10, Shimoni, 80409, Kenya; 2 Population Ecology Group, IMEDEA (UIB-CSIC) C/ Miquel Marques 21, 07190 Esporles (Balearic Islands), Spain; 3 Kenya Wildlife Service (KWS) P.O.Box 55, Ukunda, 80400, Kenya; 4 AZTI Fundazioa, Herrera Kaia, Portualdea z/g, Pasaia, Spain; 5 Centro Oceanográfico de Xixón, Instituto Español de Oceanografía (IEO), Camín de l’Arbeyal s/n, E-33212 Gijón/Xixón, Spain; Biodiversity Insitute of Ontario - University of Guelph, CANADA

## Abstract

Along the East African coast, marine top predators are facing an increasing number of anthropogenic threats which requires the implementation of effective and urgent conservation measures to protect essential habitats. Understanding the role that habitat features play on the marine top predator’ distribution and abundance is a crucial step to evaluate the suitability of an existing Marine Protected Area (MPA), originally designated for the protection of coral reefs. We developed species distribution models (SDM) on the IUCN data deficient Indo-Pacific bottlenose dolphin (*Tursiops aduncus*) in southern Kenya. We followed a comprehensive ecological modelling approach to study the environmental factors influencing the occurrence and abundance of dolphins while developing SDMs. Through the combination of ensemble prediction maps, we defined recurrent, occasional and unfavourable habitats for the species. Our results showed the influence of dynamic and static predictors on the dolphins’ spatial ecology: dolphins may select shallow areas (5-30 m), close to the reefs (< 500 m) and oceanic fronts (< 10 km) and adjacent to the 100m isobath (< 5 km). We also predicted a significantly higher occurrence and abundance of dolphins within the MPA. Recurrent and occasional habitats were identified on large percentages on the existing MPA (47% and 57% using presence-absence and abundance models respectively). However, the MPA does not adequately encompass all occasional and recurrent areas and within this context, we propose to extend the MPA to incorporate all of them which are likely key habitats for the highly mobile species. The results from this study provide two key conservation and management tools: (i) an integrative habitat modelling approach to predict key marine habitats, and (ii) the first study evaluating the effectiveness of an existing MPA for marine mammals in the Western Indian Ocean.

## Introduction

The habitats and ecosystems of the Western Indian Ocean (WIO) region hold some of the highest marine biological diversity in the world, particularly for corals and reef fish [1]. However, the increasing overexploitation of marine resources and the degradation of the habitats are threatening the marine biodiversity [2]. To mitigate these anthropogenic pressures, Marine Protected Areas (MPAs) have been used as a main management approach to protect important habitats and ecosystems including biodiversity hotspots [3]. Following this concept, the Convention on Biological Diversity (CBD) aims to improve the status of biodiversity establishing a 10% of coast and marine areas worldwide, by 2020, applying effectively and equitably managed ecologically representative and well connected systems of protected areas [4].

MPAs have been established with a variety of conservation goals, including biodiversity conservation, maintenance of genetic diversity, conservation of rare and restricted range species, prevention of overfishing [5] and enhancement of fisheries [6], among others [7]. In Kenya, there are six MPAs covering nearly 10% of the continental shelf up to 200 m depth (835 km^2^), being one of the highest percentages along the WIO [8]. These MPAs were designed initially to protect the nearshore habitats and sessile or benthic organisms [9]. After more than 20 years of MPAs establishment and monitoring, many studies have highlighted their positive impacts on local fish population (higher biomass and diversity) and status of coral reefs (higher hard coral cover and coral diversity) [10,11].

In contrast, relatively little is known about the role that MPAs play in the protection of marine top predators such as marine mammals, seabirds, and sea turtles. The growing number of anthropogenic threats that these predators are facing (*e*.*g*. fisheries bycatch), requires the implementation of urgent conservation measures to safeguard key marine areas [12]. Understanding the relationships between these highly mobile animals and their associated habitats is critical to provide the predictive power to anticipate changes in habitat use patterns and to effectively monitor and protect them. Specifically, the MPAs spatial-based conservation plans can improve population’s recovery and intensify the protection of these marine predators against threats.

Comparative studies across species and functional groups are necessary to understand the effectiveness of MPAs from a wider ecosystem-based management approach. It is important to assess whether the existing MPAs (initially established for the protection of coral reefs), also encompass key marine areas of higher trophic levels such as marine top predators. For the Kisite-Mpunguti Marine Protected Area (KMMPA), on the southern coast of Kenya, dolphins are considered flagship species. As the main attraction for the 60,000 yearly park visitors, dolphin presence is of economical importance for local communities. The Indo-Pacific bottlenose dolphin is the most abundant marine mammal species in the study area (Pérez-Jorge, unpublished data), and is currently listed as data deficient by the IUCN due to the lack of information on population abundance, habitat use, genetic diversity and population structure [13].

To identify key habitats for the population of coastal dolphins within the existing MPA, we developed species distribution models (SDM) to predict the occurrence (using presence/absence data) and abundance (combining number of sightings and group size data) of the dolphin population around the KMMPA. First, we developed a comprehensive ecological modelling approach to study the environmental factors influencing the occurrence and abundance of dolphins based on two different modelling techniques. Second, we identified recurrent, occasional and unfavourable habitats based on SDM predictions by describing those areas where dolphins are likely to occur frequently, where occurrence varies considerably inter-annually, and where no observations occur, respectively [14]. Third, we assessed the suitability of the existing MPA for the dolphin population by estimating the occurrence and abundance probabilities, as well as the percentage of recurrent, occasional and unfavourable habitats within and outside the MPA. Finally, we discuss the conservation implications of this integrated habitat modelling approach for identifying key marine areas for coastal dolphins and evaluate the effectiveness of existing MPAs.

## Materials and Methods

### Ethics Statement

This study was carried out by Kenya Wildlife Service, the government authority in the area regulating research and natural resource management. Sightings data are held by Kenya Wildlife Service. Permission for all joint Global Vision International activities was granted by the Kenya Wildlife Service Director under a 5 year Memorandum of Understanding signed in 2006. The field studies did not involve endangered or protected species, under the Kenyan Wildlife Conservation and Management Act.

### Study area and data collection

**Fig 1:**
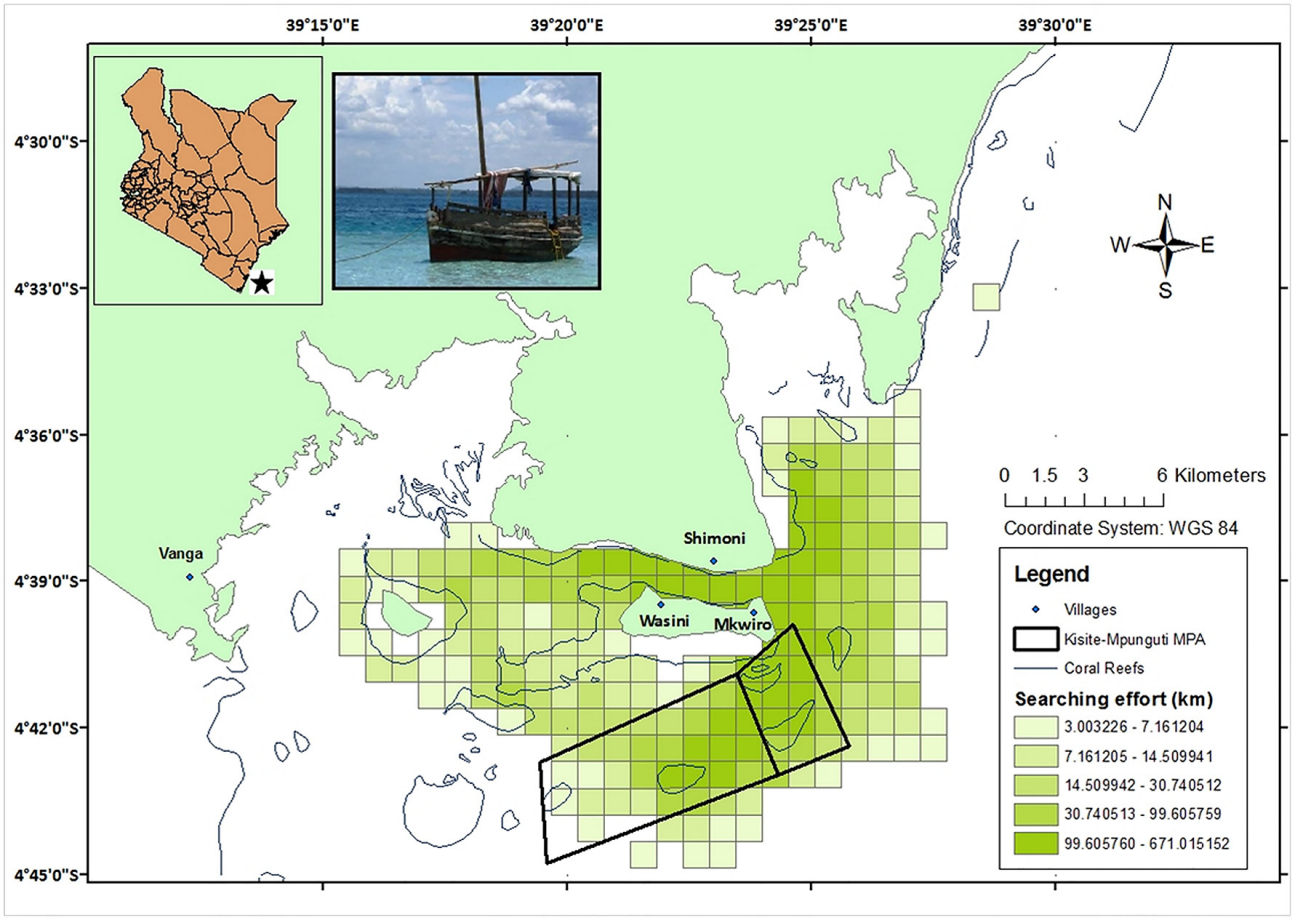
General map of the study area showing the location of the study area and an illustration of the study vessel, showing the overall survey effort (km) between 2006 and 2009, and the location of the Kisite-Mpunguti Marine Protected Area, that contains the Kisite Marine Park and the adjacent Mpunguti Marine Reserve.

Our study was focused on the southern coast of Kenya, in the Kisite-Mpunguti Marine Protected Area (KMMPA, 04°04’S—39°02’E), established in 1978. This MPA lies south of Wasini Island and incorporates the Kisite Marine Park, the largest no-take area in Kenya (28 km^2^), and the adjacent Mpunguti Marine Reserve, Kenya’s smallest reserve, artisanal fishing allowed (11 km^2^). KMMPA covers shallow waters (0–15 meters) and supports a high diversity of marine life including corals, reef fish and sea turtles (Fig 1, S1 Text).

Vessel-based surveys were conducted at an average speed of 6.9 knots all year around between January 2006 and December 2009 (except the period from January and June 2008, due to national political conflicts). Four observers scanned the water surface over 180° field of vision from the two perpendiculars to the front of the boat. Each observer covered a 45° subset of the field vision. Team members shift every 15 minutes, with an eye break after an hour of observation. Non-systematic transects were carried out during the surveys, covering an average of 69% (SD ± 15%) of the whole study area every three months, depending on climate and sea state conditions [15]. Searching effort was conducted with Beaufort sea state < 4, low swells and good visibility (≥ 1 km), reducing the probability of missing dolphins. Once animals were sighted, the research vessel approached them at low speed to identify species and to collect information on location and time of the sighting, group size and group composition.

### Data processing and exploratory analysis

Observations were standardized over a common spatial grid of 1 km by 1 km throughout the survey area using ArcMap 10.1 [16]. Survey effort was calculated on each 1 km^2^ using a UTM37S projection. Data were divided by season: summer (January to March), autumn (April to June), winter (July to September) and spring (October to December) based on local sea weather conditions. Considering the seasonal scale in the distribution of wide-ranging marine species improves model performance compared to annual averages and, in turn, we considered this temporal scale biologically meaningful [17]. Only grids with a minimum of 1km of survey effort per season and year were considered on the analysis in order to avoid small sample biases.

We transformed observations in three different quantitative ecological measurements. For each grid cell, we summed up the number of sightings and the number of individuals (i.e., group size) observed per each season and year. We recorded the number of sightings into a binary presence/absence variable by transforming into “presence” those grid cells with at least one sighting and “absence” otherwise.

### Environmental variables description and selection

We selected 10 environmental variables (5 dynamic and 5 static) based on our previous knowledge on dolphin ecology, environmental conditions in the study area and the availability of oceanographic information (Table 1, S2 Text).

**Table 1:** Description of environmental variables considered for habitat modelling, as well as their overall, absence and presence mean and range values (between brackets). The type of predictor is also described as well as their ecological interpretation.

Habitat variables	All data	Bottlenose dolphins		Predictor category	Indicative of the following processes
		Absence	Presence		
Bathymetry (BAT, m)	9.90	10.34	7.44	Static	Coastal vs. pelagic domains
	(0.12–102.12)	(0.12–102.12)	(1.66–45.68)		
Bathymetry gradient (GRAD, %)	71.40	71.14	72.83	Static	Presence of topographic features (shelf-break, seamounts)
	(3.48–100.00)	(3.48–100.00)	(12.56–99.94)		
Chlorophyll a (CHL, mg m-3)	0.61	0.63	0.48	Dynamic	Ocean productivity domains
	(0.22–1.39)	(0.22–1.39)	(0.27–1.07)		
CHL temporal change (CHLT, %)	46.59	46.79	45.43	Dynamic	Small-scale CHL variability
	(6.82–88.59)	(6.82–88.59)	(7.13–87.76)		
Sea surface temperature (SST, °C)	27.71	27.74	27.53	Dynamic	Water mass distribution
	(25.43–29.95)	(25.43–29.95)	(25.46–29.49)		
SST temporal change (SSTT, %)	10.60	10.59	10.67	Dynamic	Small-scale SST variability
	(5.71–15.74)	(5.71–15.74)	(5.80–14.79)		
Distance to coastline (COAST, km)	2.73	2.76	2.54	Static	Onshore-offshore distribution patterns
	(0.03–7.22)	(0.03–7.22)	(0.09–6.25)		
Distance to reef (REEF, km)	0.87	0.90	0.70	Static	Reef influence on dolphins diet
	(0.03–4.60)	(0.03–4.60)	(0.04–3.31)		
Distance to 100 m isobath (BATH100, km)	6.42	6.84	4.06	Static	Proximity with shelf-break (slope currents, vertical mixing and prey concentration)
	(0.19–18.44)	(0.19–18.44)	(0.63–11.61)		
Distance to oceanographic front (FRONT, km)	24.14	24.77	20.51	Dynamic	Mesoscale frontal systems
	(0.19–106.38)	(0.19–106.38)	(0.92–102.14)		

### Species distribution modelling

We used a habitat modelling approach to identify those environmental variables that most accurately described the key marine areas for dolphins within the information-theoretic approach (S1 Fig)[18].

#### Selecting environmental predictors

First, we investigated the colinearity between predictor estimating pairwise Spearman-rank correlation coefficient, which identified highly correlated variables (|rs|≥ 0.7), previously standardized [19] (S1 Table). Second, to keep the most explanatory environmental variable we ran Generalized Linear Models (GLMs) to check which of these pairs of variables better explained the observed response variable using the Akaike Information Criteria (AIC) value, using only one predictor at a time. The model with a lower AIC value explained better the response variable. The same procedure was applied for each of the three ecological measurements (presence-absence, sightings and group size data. This led to the removal of GRADIENT and CHLA.

#### Model construction

We used Generalized Linear Models (GLMs) and Generalized Additive Models (GAMs) to examine the relationship between response variables and explanatory variables. In the case of occurrence data, we developed logistic regressions using a binomial distribution and logit link function. Number of sightings and group size were modelled following a negative binomial distribution. This model was selected over the Poisson distribution since the latter showed overdispersion in the null model. Additionally, the number of kilometres per grid (i.e., survey effort) was included as an offset term, therefore preventing from possible biases produced by uneven sampling. In GAM models, the smoothing splines were limited to a maximum of 3 degrees of freedom to capture non-linear associations without increasing the complexity of the functions towards unrealistic conclusions [20]. Models were built within the R environment (version 2.15.3;[21]) using ‘MASS’ [22] and ‘mgcv’ packages [23].

#### Model selection and multimodel inference

We implemented the information-theoretic approach to evaluate competing models by assessing their relative support in relation to observed data, rather than using the best single model approach [24]. Models were constructed for all possible combinations of explanatory variables and then ranked depending on the support of each of these models using the AIC values and the Akaike weight [24]. The Akaike weight of each model is the relative likelihood of that model compared with the remaining models and was used to identify the 95% confidence set of models. To identify the 95% confidence set, we selected the model with the highest Akaike weight and added the models with the next highest weights until the cumulative Akaike weights > 0.95. When the model with lowest AIC value has an Akaike weight value lower than 0.9, a model averaging procedure might be more appropriate to account for model and parameter uncertainty [24]. The model averaged predictions were expected to be more robust than those from single best model approach. Averaged coefficients were estimated using the MuMIn package [25]

#### Model checking

Species distribution data are characterised by spatial autocorrelation since distribution data in close location are more similar than would be expected in randomly distributed data [26]. Significant spatial autocorrelation can invalidate the common assumption that observations are independent, and identify spurious significant relationships (Type I error) [27]. Spatial autocorrelation was checked on the residuals of the model with the lowest AIC using the Moran’s I index [28] and spatial correlograms with the ‘ncf’ package [29]. The Moran’s I index ranges from -1 (negative autocorrelation—perfect dispersion) to +1 (positive autocorrelation—perfect correlation), with values around zero being indicative of random spatial patterns [28]. The spatial correlogram estimate the spatial dependence through testing significance within each distance class by a randomization test [30]. We did not include any spatial autocorrelation structure in our models since we did not find significant spatial autocorrelation (S2 Table).

#### Model evaluation

A crucial stage of the SDMs is to determine the predictive ability of final models to assess their applicability in conservation and management programmes. To this end, we used a cross-validation procedure to evaluate the accuracy of final models. Models were built with the 70% of the original data (training data: 2006, 2007 and 2008) and evaluated on the remaining 30% (test data: 2009). The predictive performance of models was measured through the concordance index (*C-index*) with the R package ‘Hmisc’ [31]. The *C-index* is applicable to continuous and categorical data, as the predictive discrimination is related to a rank correlation between predicted and observed outcomes [32]. This index is identical to the most widely used measures for model discrimination, the area under the Receiver Operating Characteristic curve (AUC) [33]. The *C-index* ranges from 0 to 1 and models with values from 0.7 onwards are considered with good discrimination ability (0.7–0.8: ‘moderate discrimination’, 0.8–0.9: ‘good discrimination’; 0.9–1: ‘excellent discrimination’) [34].

#### Ensemble predictions

Averaged models of GLMs and GAMs were combined to produce an ensemble prediction since the accuracy of SDMs predictions could be improved by applying consensus methods [35–38]. The weighted average (WA) consensus method was used to create the ensemble predictions from single-model predictions assigning weights to each model and using the pre-evaluated *C-index*, as follows [38]: WA_i_ = Σ_j_ (*C-inde*x_mji_ x mj_i_)/ Σ_j_ *C-index*_mji_, where mj_i_ are the probability-of-occurrence of values of the *i*th model in a given grid cell for the j-selected single- models for which pre-evaluation *C-index* values were the highest.

### Identifying key marine areas for dolphins to measure the influence of the existing MPA

To identify priority marine areas for dolphins in southern Kenya, we predicted the spatial distribution of the three ecological measurements through maps of 1x1km resolution with R. We extracted seasonal predictors from 2006 to 2008, training data, and applied the 95% of confidence set of models to forecast the occurrence, sightings and group sizes distributions. From this, we obtained an average prediction of the study area and calculate the standard deviation (SD) to measure the stability of the predicted distribution, with stable and unstable habitat represented by low and high SD [39]. We combined these predictions to define three categories of distribution areas [14] (1) recurrent areas, where dolphins are frequently observed every year, represented by grid cells with high mean (higher than the average mean across all grids cells and study years) and low SD (lower than the average SD across all grid cells and study years); (2) occasional areas, where dolphins’ presence varies from year to year, represented by grid cells with high SD (higher than the average SD across all grid cells and study years); and (3) unfavourable areas, where dolphins are almost never seen, represented by grid cells with low mean (lower than the average mean across all grid cells and three years) and low SD (lower than the average SD across all grid cells and three years). Recurrent and occasional cells were used to define key areas for dolphins.

Finally, to evaluate how the existing MPA encompasses key habitat areas we compared the percentage of each category of distribution areas and mean predictions inside and outside the MPA for each of the occurrence and abundance models, and applied sequential t tests [14].

### Abundance estimates

The ensemble predictions obtained for sightings and group size on each grid cell were multiplied to predict abundance of dolphins [40]. As we did not apply line transect methodologies [41], we calculated an approximate effective sampling width with the distance and angles data from the 2008–2009 sightings (those data were not recorded for the 2006–2007). The average effective sampling width was 92 m (SD ± 92). Thus, we assumed that we missed only a small part of dolphins’ sightings. The total number of dolphins on the study area was obtained by summing the previously predicted abundance of all the grid cells.

## Results

We conducted a total of 551 dedicated vessel-based surveys between 2006 and 2009 (Table 2). Overall, dolphins were present in 77 of the total 194 surveyed grid cells, with an average of 2.53 (SD ± 5.48) sightings and 23.26 (SD ± 52.22) individuals per group. During the surveys, dolphins were mainly encountered on the east side of the study area, with the highest number of sightings and group sizes within and around the MPA, and the lowest in the North-East side of the survey area (S2 Fig).

**Table 2:** Searching effort per year and numbers of the three ecological measurements.

Year	Seasons	Searching effort (Km)	Number of grid cells present	Sightings	Group size
2006	4	3887	73	131	981
2007	4	3757	89	137	1184
2008	2	1849	42	70	747
2009	4	4009	94	152	1601
**Total**	**14**	**13502**	**298**	**490**	**4513**

### Modelling ecological measurements

To estimate the average models and reduce models uncertainty, a higher number of models were combined to achieve the 95% confidence set in GAMs compared to GLMs (Table 3). Likewise, GAM showed a higher deviance explained on the best model than GLMs. In terms of the GLM output, BATH100 and FRONT were the predictors with the strongest negative effect, showing the highest probabilities close to the 100 meter isobaths and oceanic fronts for all three ecological measurements (S3 Fig), as well as SST in a minor degree. In the case of GAM, REEF and FRONT were the most important variables describing the three ecological measurements; with higher probabilities occurring in close proximity to the reefs and frontal systems, and in shallow waters relatively close to the shelf-break (S3 Fig, see S3 Text for further explanation on the modelling results). Sightings were influenced by small temporal variations on chlorophyll among seasons. In addition, group size showed an increase during the season with low temperatures. Regarding model evaluation, the ensemble predictions yielded the best model performance for all three ecological measurements (note C-index values >0.8; Table 3).

**Table 3:** Summary of the habitat modelling output and model evaluation.

Ecological index	Model	ED from MwlAIC		# variables in MwLAIC	Number models in 95CS	TRAIN DATA		TEST DATA	
		TRAIN DATA	TEST DATA			Mean C-index	SD C-index	Mean C-index	SD C-index
Presence/absence	GLM	9.84	6.28	3	52	0.86	0.03	0.85	0.04
	GAM	17.50	19.00	5	13	0.81	0.03	0.78	0.04
	Ensemble	NA	NA	NA	NA	0.87	0.03	0.84	0.04
Sightings	GLM	15.27	10.20	3	45	0.85	0.03	0.84	0.03
	GAM	27.00	31.10	6	10	0.79	0.03	0.78	0.04
	Ensemble	NA	NA	NA	NA	0.86	0.02	0.84	0.03
Group size	GLM	15.14	15.81	4	102	0.82	0.03	0.81	0.03
	GAM	28.60	41.30	7	7	0.77	0.03	0.75	0.04
	Ensemble	NA	NA	NA	NA	0.84	0.03	0.81	0.04

ED: explained deviance. MwlAIC: Model with Lowest Akaike’s Information Criteria (AIC). 95CS: 95% confidence set. C-index: concordance index.

### Dolphin abundance estimations

The estimated total abundance for the 2006–2008 period was 91.54 ± 55.32 dolphins, with an average predicted abundance of 0.83 ± 1.74 dolphins/km^2^. The highest abundance predictions occurred within the MPA with 54.85 ± 40.75 dolphins compared to the 36.69 ± 18.30 dolphins outside the MPA.

### Key areas for dolphins

**Fig 2:**
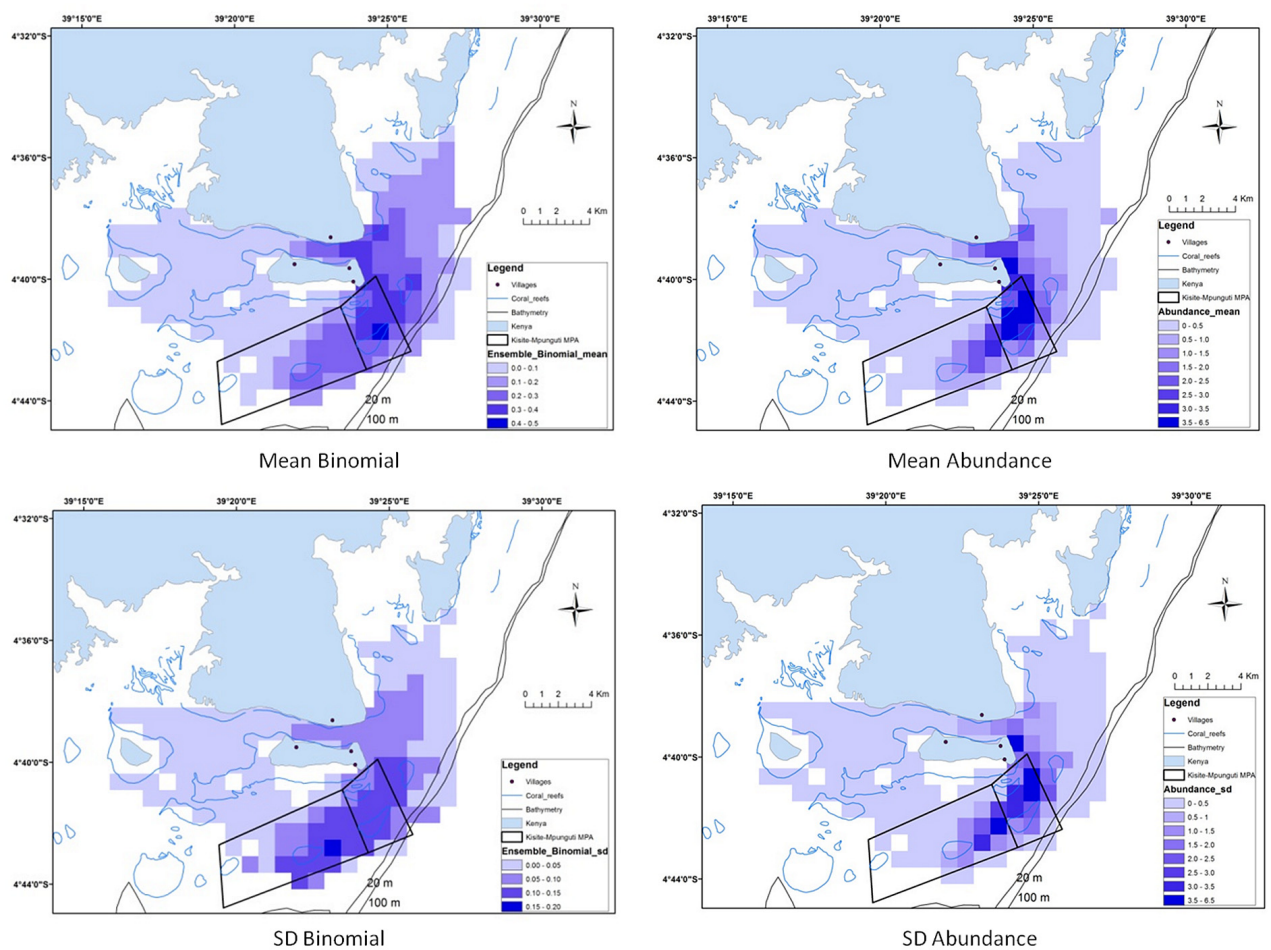
Distribution maps of the binomial and abundance predictions (Mean and SD) over the 2006 and 2008 period (training data)

**Fig 3:**
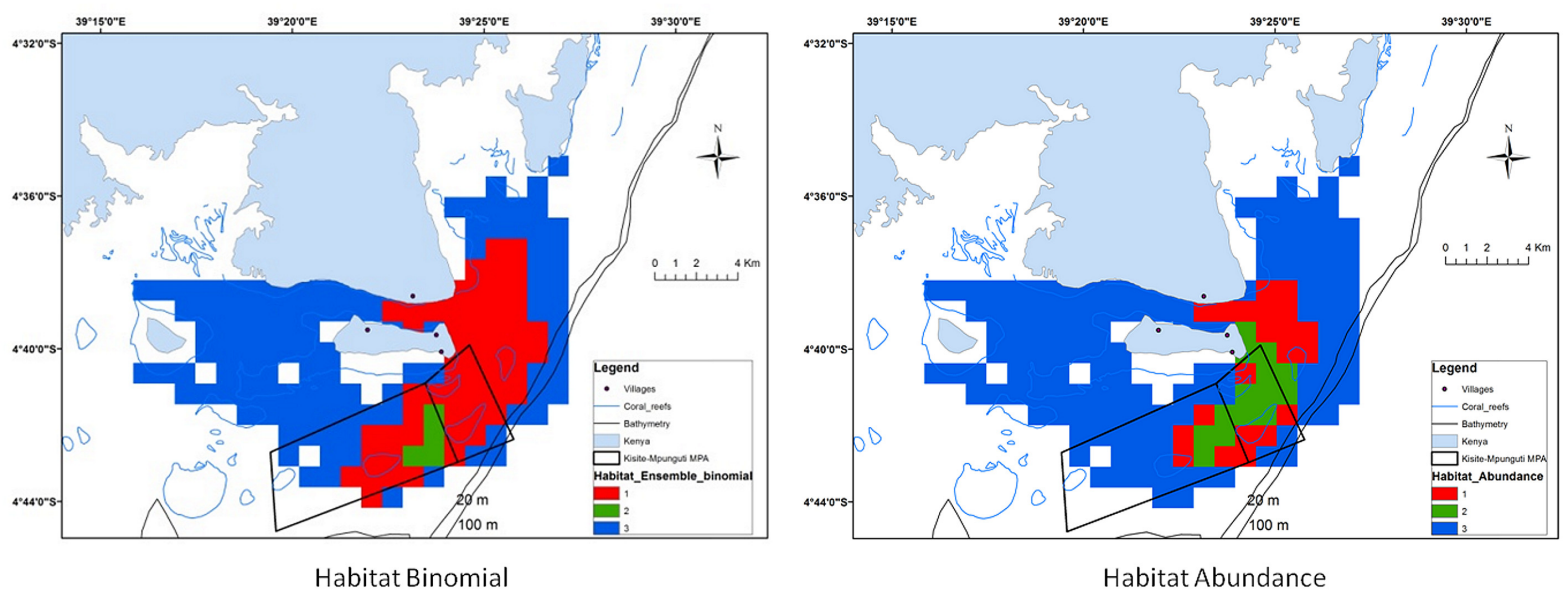
Type of habitats for the binomial and abundance predictions over the 2006–2008 period (training data). (1) recurrent areas, (2) occasional areas; and (3) unfavourable areas.

**Fig 4:**
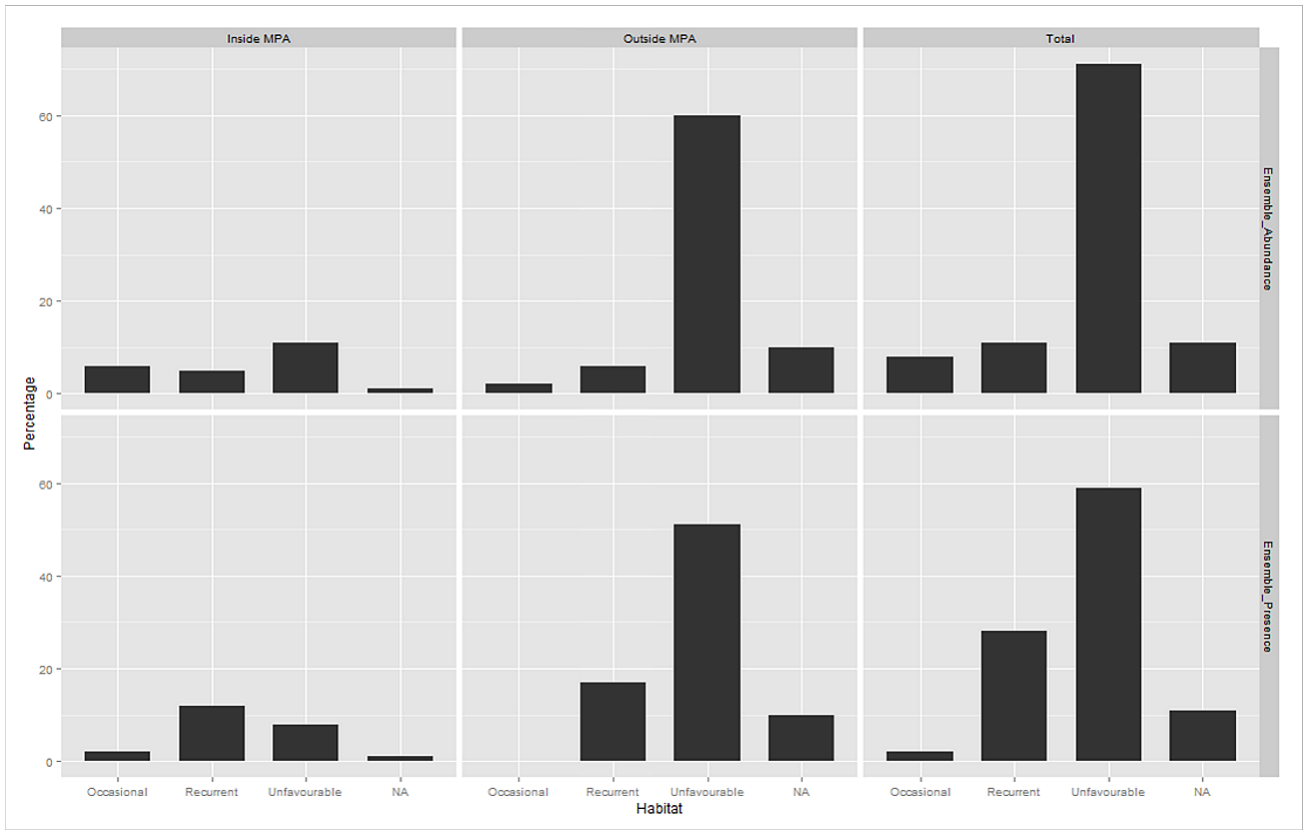
Percentage of areas with recurrent, occasional and unfavourable habitats inside and outside the MPA for the predicted ensemble binomial and abundance. A 10.52% of grid cells have no category due to the lack of sampling during certain periods.

**Fig 5:**
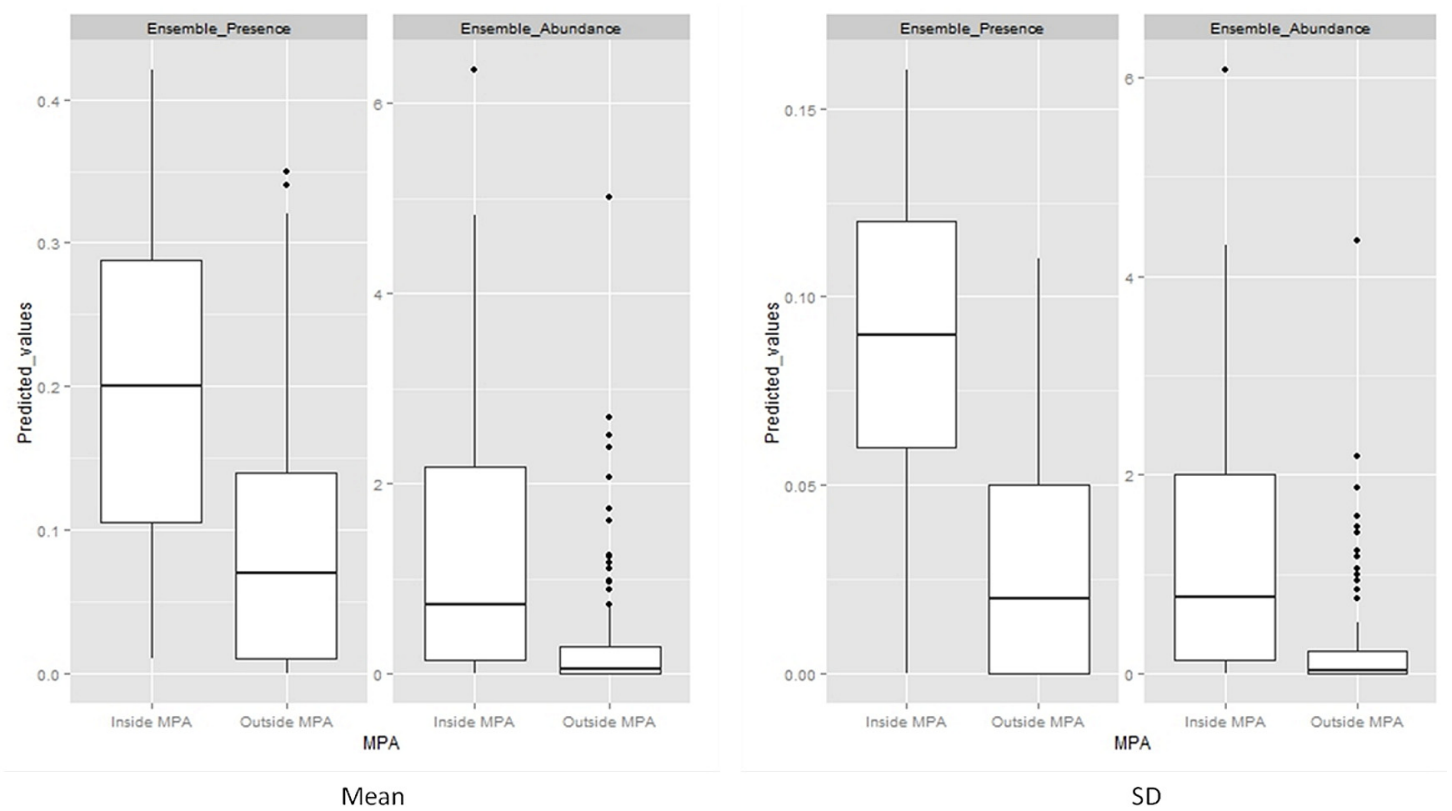
Mean and SD of the binomial and abundance predictions (median, 25–75%, inter-quartile range, non-outlier range, and outliers) in relation to the MPA (inside-outside).

Our model predictions matched observed patterns within the range of the dolphins and identified important distribution areas on the east side of the study area (Fig 2). The MPA was identified as a critical area for all three ecological measurements, with high variability (SD) in predictions probably due to seasonal variations (S4 Fig). The occurrence models predicted the highest dolphin probabilities within the MPA and on the east side of Wasini Island (Fig 2). For abundance models, predicted maps matched the key areas identified by the occurrence models, but the maximum abundances were concentrated on a smaller area (Fig 2). In this case, the highest probabilities were mostly encountered within the MPA. The combination of the previous predictions maps resulted in the identification of recurrent, occasional and unfavourable habitats (Fig 3). The ensemble predictions of the occurrence and abundance models defined a 59% and 71% of unfavourable habitats within the study area respectively, and a total of 31% and 18% combining recurrent and occasional habitats for each ensemble prediction (Fig 4) However, an important percentage (47%) of these recurrent and occasional areas was identified inside the MPA using presence-absence models and a 57% using abundance models. Moreover, presence probability and abundance predictions were significantly higher within the MPA than outside (for occurrence predictions t = -6.622, P>0.05; for abundance predictions t = -6.618, P>0.05), showing the higher preference for these areas (Fig 5).

## Discussion

### Spatial ecology of coastal dolphins

As a result of our integrative ecological modelling approach, we ascertain that both static and dynamic variables influenced the spatial ecology of the *T*.*aduncus*. Our modelling showed that the reefs inside the MPA and along the east side of Wasini Island are selected by coastal dolphins. This habitat preference is supported by previous studies on the foraging ecology of the species, that found *T*. *aduncus* to feed upon inshore reef fish and cephalopods [42]. In addition to reef prey, species comprised within the *Tursiops* genera feed upon alternative prey such as bathydemersal fish and cephalopods in offshore waters along the WIO [43]. Thus, the strong influence of the isobaths of 100m on dolphin spatial ecology would likely indicate that they exploit not only inshore (shallow waters) but also offshore waters (proximity to deep waters) feeding on different prey.

In addition to static features, the spatial ecology of coastal dolphins was strongly affected by dynamic variables such as distance to frontal systems, when oceanographic fronts are closer to shore. Oceanographic fronts are important features due to their intense mesoscale activity where processes of upwelling/downwelling take place that enhance marine productivity, leading to the formation of predictable prey patches [44]. Other dynamic variables that played a secondary role on driving sightings and group size patterns were SST and CHL. Several studies have linked cetacean habitat preferences to dynamic variables, showing the effect of these predictors to define the species distribution [15,45].

### Transferring modelling outputs into a MPA context

Species distribution models (SDM) are the first essential step to understand the influence of environmental drivers on the spatial distribution of a given species. Previous studies have shown that non-parametric regression methods, such as GAMs, had better predictive performance than parametric methods, such as GLMs [46]. In comparison, our results yield slightly higher predictive performance for GLMs than GAMs for the three ecological measurements. Despite these minor differences in predictive performance, both techniques provided very different spatial predictions, probably due to the underlying assumptions relationship between the modelling technique and the environmental predictors [47]. Additionally, comparing the predictions from all models revealed that occurrence and sightings models had marginally better discrimination ability than group size models. The accuracy of the predictive models was improved by applying consensus methods and combining them into an ensemble model, reducing also the uncertainty from the most traditional approach of selecting the best model from an ensemble of forecasts [36]. Another way of minimising model uncertainty was through the multimodel inference based on the information-theoretic approach. To our knowledge this is the first time that ensemble models and model averaging are used to predict marine mammal habitats.

To develop effective conservation science, modelling outputs have to be discussed directly within the context of a MPA. For instance, this study highlights the association of coastal dolphins to static as well as dynamic oceanographic variables, revealing the need to incorporate dynamic and spatially explicit conservation actions for marine top predators [48]. Recent conservation planning demands a shift to more dynamic and adaptive management of marine resources to adjust to the current challenges facing the marine environment and marine species [49]. While it is important to consider dynamic marine features (*e*.*g*., eddies, fronts) to identify pelagic biodiversity hotspots for the establishment of dynamic MPAs [50], we need to recognize that those MPAs pose a management challenge compared to static systems established as permanent closures [51].

### Conservation implications: the role of existing MPA for coastal dolphins

An effective ecosystem-level management of a MPA depends acutely upon the quality of information available, not only for delineating boundaries but also to understand how these areas are used by animals and which components influence their distribution and abundance [52]. Our modelling output evidences the positive benefits of the existing MPA for coastal dolphins, whose occurrence and abundance were higher within this spatial-based management unit than outside. In addition, there is evidence that other taxonomic groups have benefited from the establishment of this and other MPAs along the coast of Kenya. For instance, abundance and biomass of coral reef fish have significantly increased since the establishment of the existing MPA [53]. While the use of presence-absence data determined 31% of the total study area as recurrent and occasional areas, the abundance data reduced to only 18% of the area. This shows the hierarchical patterns of distributions, with localised areas of high relative abundance nested within the distribution area used by the species in the study area [54]. Nevertheless, 53% and 43% of recurrent and occasional areas occurred outside the MPA using presence-absence and abundance models respectively, suggesting that MPA does not encompass the whole ecological needs of dolphins. Finally, more than 65% of our study area was defined as unfavourable for Indo-Pacific bottlenose dolphins, probably due to multiple factors such as non-optimal environmental conditions, evolutionary strategies to reduce competition with other closely related species (*e*.*g*., *Sousa chinensis* [55]) and intensive fishing (Pérez Jorge, unpublished data).

An optimal design of an MPA expected to protect a population would include the entire year-round distribution of that population [56]. Although the design for some resident or non-migratory species may be possible to achieve, the protection of highly migratory or mobile species present a major challenge for spatial management. Thus, when only a small portion of a population’s range can be included within a MPA, it is crucial to protect critical habitats for the species’ survival (*e*.*g*., breeding and foraging areas) where they are particularly vulnerable to anthropogenic impacts [12]. This study determined that the area encompassed by the MPA is certainly insufficient to satisfy the spatial requirements of the species, not covering a high percentage of the recurrent areas that constitutes critical habitat for vital activities every year. However, areas containing critical habitat outside the MPA are partially incorporated in the proposed collaborative co-management initiative introduced by the Kenyan government in 2006 [8]. Co-management areas are developed and enforced by local bylaws with respect to the use of and access of fisheries. Early findings suggest that they increase fish biomass if an effective compliance takes place [57]. Nevertheless, this will require further investigation due to the recent implementation of these co-management initiatives.

MPAs have been advocated for the conservation of marine mammals, but few examples have empirical evidence that they are effective [58]. Quantifying the effects of MPAs is crucial to evaluate their efficiency as management tools and the protection of the species [59]. The results from this study suggest that Kisite-Mpunguti MPA represents an important area that seemingly encompasses key habitat features of ecological and behavioral importance to the Indo-Pacific bottlenose dolphins, and it should be considered as a critical habitat for the species which requires special management considerations. This species is important ecologically, as a potential indicator species which protection may ensure the health of other key elements of the marine ecosystem, and economically, through the growing dolphin-watching industry [12]. It has been shown that dolphin tourism can have negative impacts on dolphin populations, especially when not monitored or unsustainably managed [60]. Impacts may be long-term and life-threatening; both at the individual and population level [61]. Other anthropogenic impacts on the cetacean populations such as overfishing [62] and seismic exploration [63] have recently been identified as the main threats for marine mammals around Kisite-Mpunguti MPA [64]. Especially taking into account the restricted inshore habitat of the species, it is important to evaluate the effectiveness of the code of conduct implemented by Kenya Wildlife Service in 2007 as well as, to assess the other identified threats.

## Conclusions

We provide two key conservation and management tools: (i) an integrative habitat modelling approach to predict key marine habitats, and (ii) the first study evaluating the effectiveness for marine mammals of an existing MPA in the WIO. Our results show how the modelling technique selection may influence the identification of key marine areas, and how using ensemble models can improve the predictive performance, successfully predicting areas of importance of a given species. We recommend the use of these robust ensemble models for decision makers in designing and identifying MPAs. In the case of coastal dolphins, these ensemble predictions forecast a higher occurrence and abundance of dolphins within the MPA, covering a large percentage of recurrent and occasional areas (47% and 57% using presence-absence and abundance models respectively), but does not adequately protect all of them. We propose to extend the protection to incorporate all occasional and recurrent areas, which are critical habitats for the species. MPAs not only benefit fish and invertebrate populations, but also improve the prey base for top marine predators and reduce their threats through spatial protection [12,56]. We highlight the need to analyze the level of actual protection of existing MPAs as it may not provide the proper representation for upper-trophic level species. Finally, this study could also be applied to evaluate the potential effects on the distribution and abundance of top marine predators within a global change scenario, taking into account that climate change will affect the distribution and availability of prey in the short and long term [65].

## Supporting Information

S1 Fig Workflow of the habitat modelling procedure applied. (TIF)

S2 Fig Observed distributions of all three ecological measurements. (TIF)

S3 Fig Functional relationship between dolphins predicted probability and predictors based on GLM and GAM output. (TIF)

S4 Fig Mean and SD predictions probability over the years 2006 and 2008. (TIF)

S1 Table Pair-wise correlation between predictor variables by means of Spearman-rank correlation coefficient. (TIF)

S2 Table Model checking results (Moran’s I index values). (TIF)

S1 Text Description study area. (DOCX)

S2 Text Environmental variables description and selection. (DOCX)

S3 Text Modelling results from the three ecological measurements. (DOCX)
