# Supplementary figures and images for: Can Static Habitat Protection Encompass Critical Areas for Highly Mobile Marine Top Predators? Insights from Coastal East Africa
Source: PLoS One. 2015 Jul 17;10(7):e0133265. doi: 10.1371/journal.pone.0133265 (PMC4506016; doi:10.1371/journal.pone.0133265)

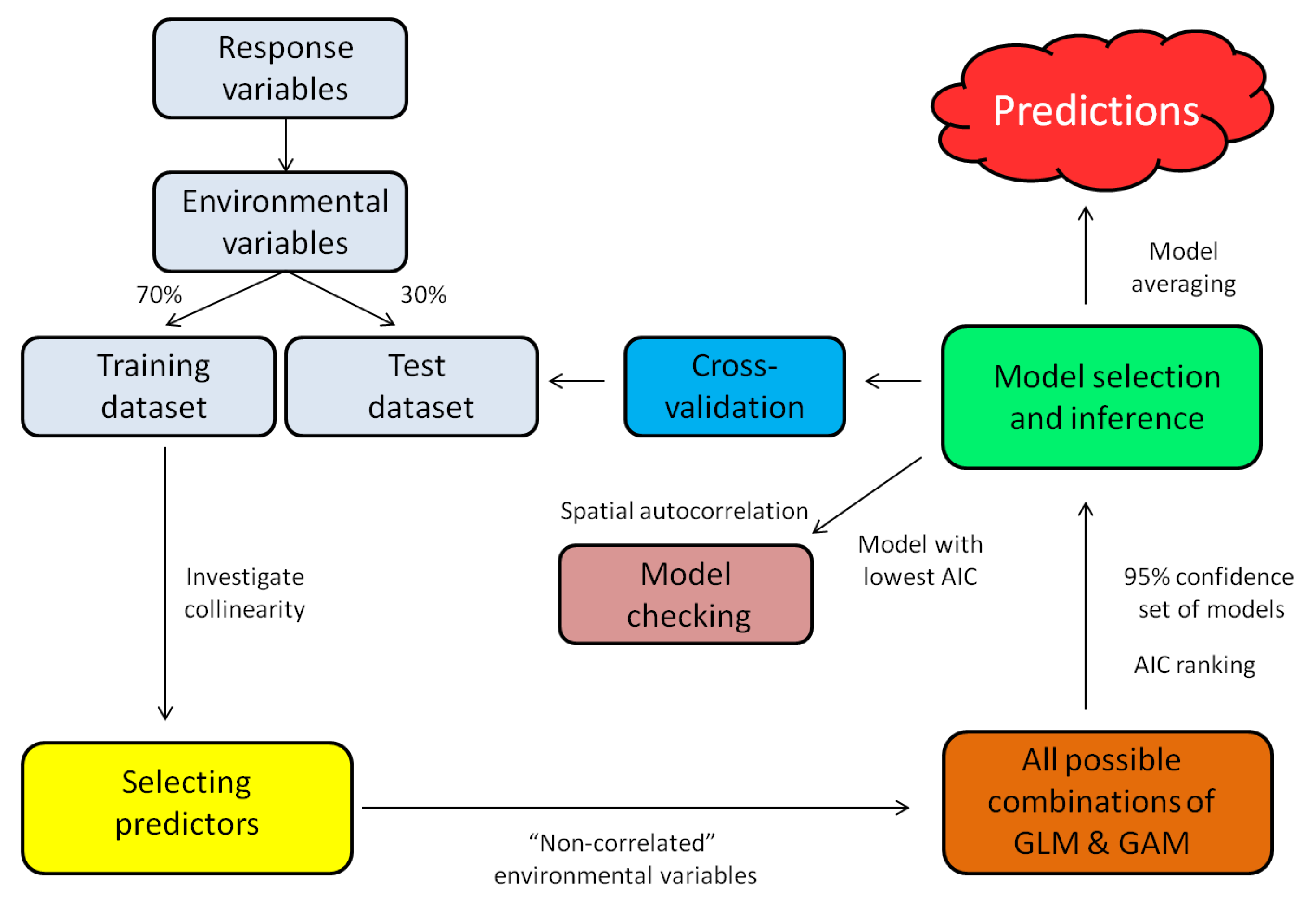

Supplement: S1 Fig — (TIF) [file pone.0133265.s001.tif]

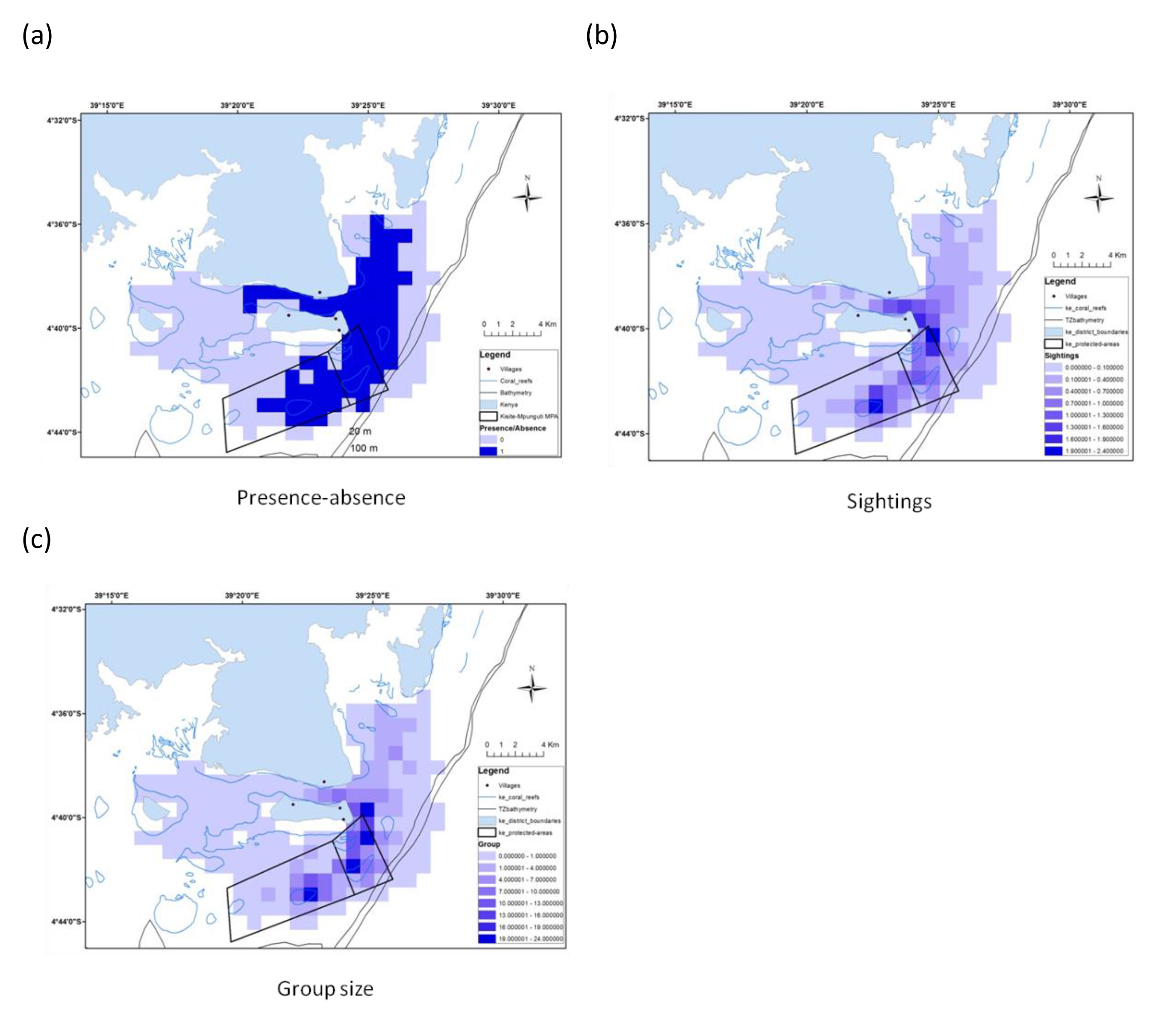

Supplement: S2 Fig — (TIF) [file pone.0133265.s002.tif]

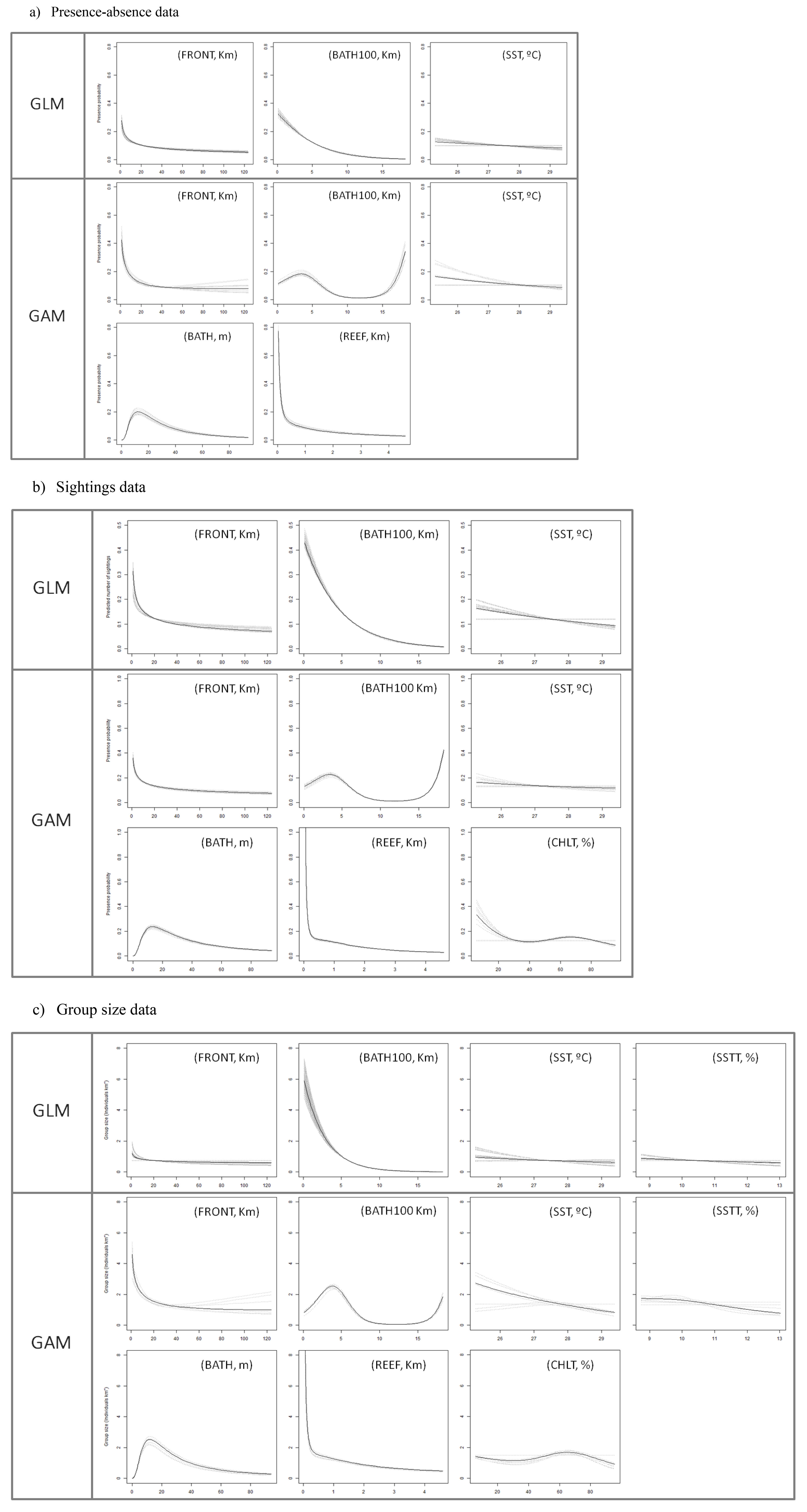

Supplement: S3 Fig — (TIF) [file pone.0133265.s003.tif]

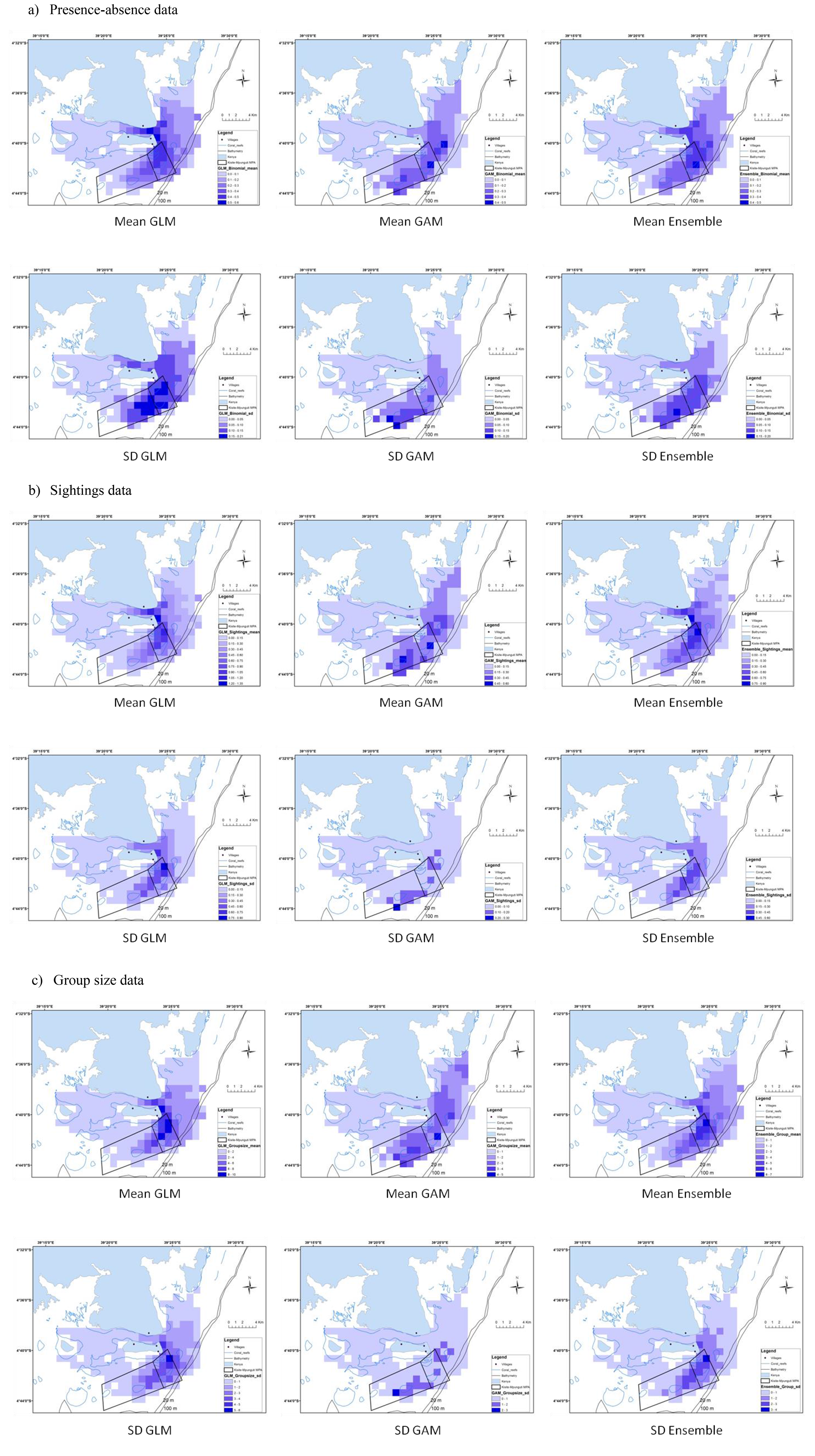

Supplement: S4 Fig — (TIF) [file pone.0133265.s004.tif]

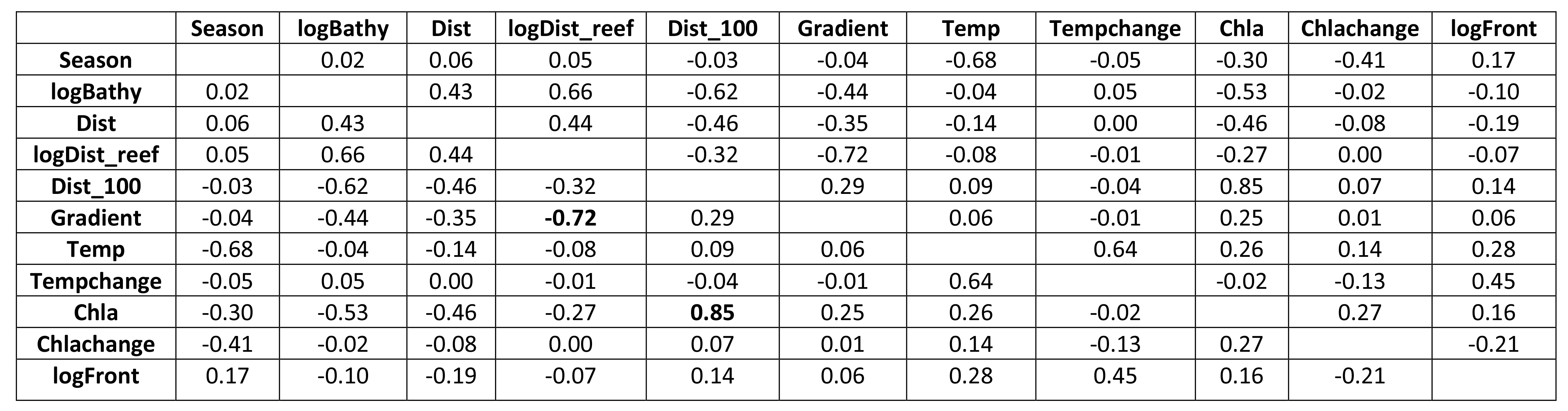

Supplement: S1 Table — (TIF) [file pone.0133265.s005.tif]

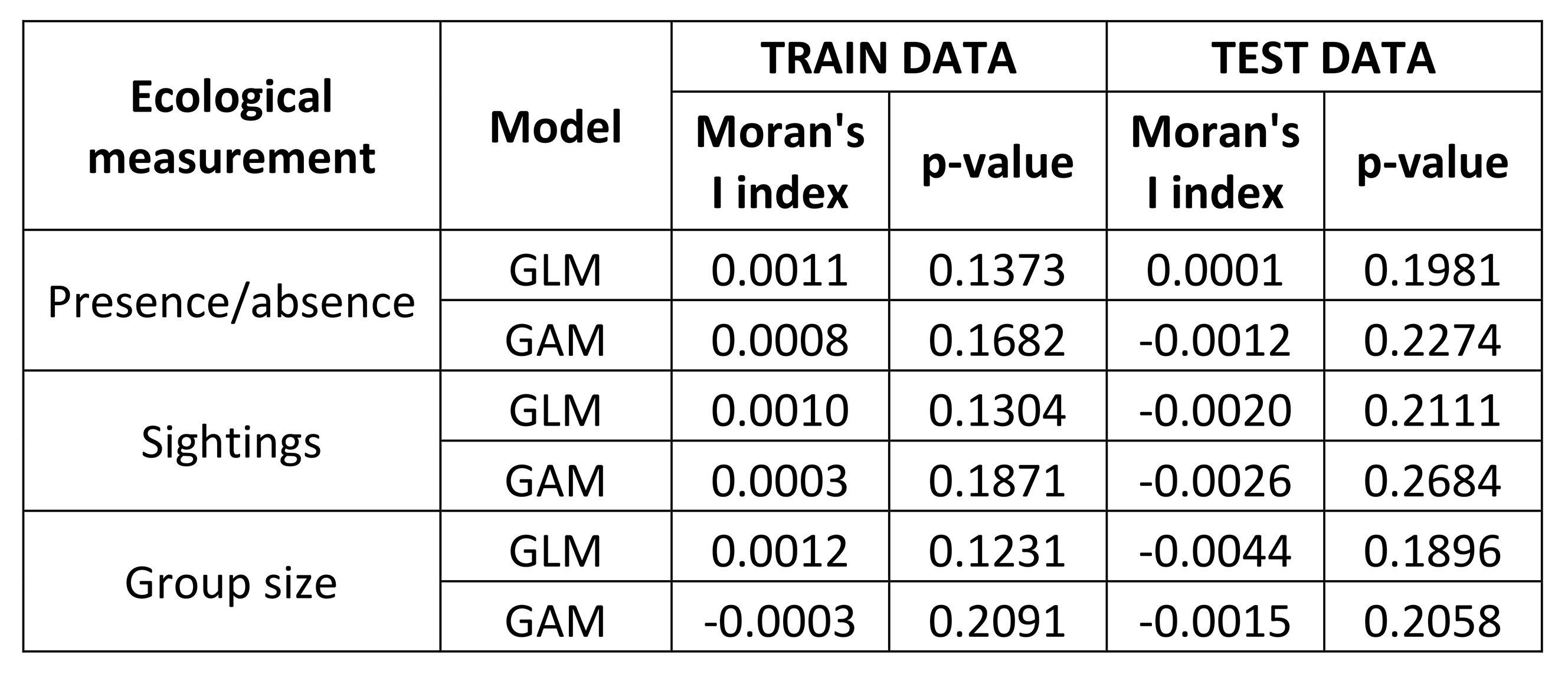

Supplement: S2 Table — (TIF) [file pone.0133265.s006.tif]
