# Supplementary material for: Can Static Habitat Protection Encompass Critical Areas for Highly Mobile Marine Top Predators? Insights from Coastal East Africa
Source: PLoS One. 2015 Jul 17;10(7):e0133265. doi: 10.1371/journal.pone.0133265 (PMC4506016; doi:10.1371/journal.pone.0133265)
Supplement: S1 Text — (DOCX) [file pone.0133265.s007.docx]

Text S1

**Description study area**

In Kenya, there are six Marine Protected Areas spread along the coastline of Kenya, including four fisheries closure marine parks and six restricted fishing marine reserves, covering nearly 10% of the continental shelf up to 200 m depth, being one of the highest percentages along the Western Indian Ocean [1–3]. Our study was focused on the southern coast of Kenya, more specifically in the Kisite-Mpunguti Marine Protected Area (KMMPA, 04°04’S - 39°02’E). KMMPA covers shallow waters (0-15 meters) and supports a high diversity of marine life including corals, reef fish and sea turtles. This MPA lies south of Wasini Island and incorporates the Kisite Marine Park, the largest no-take area in Kenya (28 km²), and the adjacent Mpunguti Marine Reserve, Kenya’s smallest reserve, artisanal fishing allowed (11 km²) (Fig. 1). This MPA was gazetted in 1978 and it is managed by the Kenya Wildlife Service (KWS) since 1988. KWS is a government parastatal institution with the aim of conserving Kenya’s wildlife and it is responsible for the management of all protected areas around the country.

Furthermore, the MPA protects islands such as Lower and Upper Mpunguti, which are home to populations of the rare coconut crab (*Birgus latro*), and Kisite, which has been considered an Important Bird Area (IBA) hosting species such as the dimorphic egret *Egretta dimorpha*, the sooty tern *Sterna fuscata* and large numbers (up to 1,000 breeding pairs recorded) of roseate terns *Sterna dougallii* (BirdLife International 2014). Of equal importance for the long-term environmental management of this region are the habitats and wildlife in areas surrounding the MPA that do not fall under protection, and encompasses a wide range of habitats from mangrove forests, coral reefs, seagrass beds and offshore waters which are considered important fish nursery grounds.

The study area is influenced by the monsoon winds of the Indian Ocean. The north-eastern monsoon (known as *kaskazi*) blows from December to March, bringing calm weather, with low wave height and temperatures comprise between 28-32ºC. The south-eastern monsoon (known as *kusi*) blows from May to October, usually windy, rough seas and cool temperatures (24-26ºC). The transition periods are characterized by variable and weaker winds. Rainy periods occur between the monsoon seasons with the long rains occurring from March to May and the short rains from October to December, with a mean annual rainfall ranging from 1000 to 1600mm [4,5].

**References**

1. McClanahan TR, Mwaguni S, Muthiga N a. (2005) Management of the Kenyan coast. Ocean & Coastal Management 48: 901–931. doi:10.1016/j.ocecoaman.2005.03.005.

2. Wells S, Burgess N, Ngusaru A (2007) Towards the 2012 marine protected area targets in Eastern Africa. Ocean & Coastal Management 50: 67–83. doi:10.1016/j.ocecoaman.2006.08.012.

3. Rocliffe S, Peabody S, Samoilys M, Hawkins JP (2014) Towards A Network of Locally Managed Marine Areas (LMMAs) in the Western Indian Ocean. PloS one 9. Available: http://www.ncbi.nlm.nih.gov/pubmed/25054340. Accessed 24 July 2014.

4. Camberlin P, Philippon N (2002) The East African March–May Rainy Season: Associated Atmospheric Dynamics and Predictability over the 1968–97 Period. Journal of Climate 15: 1002–1019.

5. Mutai CC, Ward MN (2000) East African Rainfall and the Tropical Circulation/Convection on Intraseasonal to Interannual Timescales. Journal of Climate 13: 3915–3939.
