# Supplementary material for: Can Static Habitat Protection Encompass Critical Areas for Highly Mobile Marine Top Predators? Insights from Coastal East Africa
Source: PLoS One. 2015 Jul 17;10(7):e0133265. doi: 10.1371/journal.pone.0133265 (PMC4506016; doi:10.1371/journal.pone.0133265)
Supplement: S2 Text — (DOCX) [file pone.0133265.s008.docx]

Text S2

**Environmental variables description and selection**

Regarding static variables, robust correlations between water depth and many species of marine mammals have been found in different regions and ocean basins [1,2], thus making bathymetry (BAT) an appropriate variable as an environmental proxy for habitat models. To account for spatial gradients of depth, we estimated bathymetric gradients (GRAD) within every grid cell using the following formula: [(maximum depth - minimum depth)*100]/maximum depth. In addition, we also estimated the closest distance to the fronts (FRONT), coast (COAST), reef (REEF) and 100 meter isobaths (ISOBATH) from the centroid of each grid cell. BAT, REEF and FRONT values were log-transformed because the minimum and maximum values differed by an order of magnitude.

Regarding dynamic variables, we included sea surface temperature (SST) and chlorophyll *a* density (CHL) since marine mammals are believed to adapt to specific temperature regimes, and to associate with highly productive areas (as indicated by high chlorophyll a values). Monthly composite for SST and CHL were derived from aqua-MODIS sensor and converted with the Marine Geospatial Ecology Tool (MGET, [3]) from their original formats to raster formats compatible with ArcGIS. As the selected unit of observation (1 km) was at a finer spatial resolution than the remotely sensed habitat variables (4 km), SST and CHL were interpolated using the ordinary kriging function in ArcMap (Geostatistical Analyst). We performed an exploratory screening to ensure that interpolated spatial patterns matched original patterns by means of semivariogram output and visual inspection. Subsequently, as SST and CHL were not normally distributed the median was used as the central tendency instead of the mean for each season within every 1 Km^2^ grid cell. In addition, we estimated a temporal gradient for every season to account for the small scale variability of dynamic variables as follows: SSTT and CHLT = [(maximum value - minimum value)*100]/maximum value, with maximum being the highest and minimum the lowest monthly value for the six month period comprising the three months prior to the season and the actual season [4].

Finally, marine top predators are associated with oceanographic fronts as they find favourable feeding conditions and this will likely influence their distribution and abundance [5]. MGET’s Cayula-Cornillon Fronts tool was used to identify these fronts by detecting the edge of adjacent water masses of different SST with the Cayula-Cornillon algorithm [6], using the SST images from AVHRR Pathfinder SST dataset [7].

**References**

1. Moore SE, Waite JM, Friday NA, Honkalehto T (2002) Cetacean distribution and relative abundance on the central-eastern and the southeastern Bering Sea shelf with reference to oceanographic domains. Progress in Oceanography 55: 249–261.

2. Baumgartner MF, Mullin KD, May LN, Leming TD (2000) Cetacean habitats in the northern Gulf of Mexico. Fishery Bulletin 2: 219–239.

3. Roberts JJ, Best BD, Dunn DC, Treml E a., Halpin PN (2010) Marine Geospatial Ecology Tools: An integrated framework for ecological geoprocessing with ArcGIS, Python, R, MATLAB, and C++. Environmental Modelling & Software 25: 1197–1207. Available: http://linkinghub.elsevier.com/retrieve/pii/S1364815210000885. Accessed 17 July 2014.

4. Oppel S, Meirinho A, Ramírez I, Gardner B, O’Connell AF, et al. (2012) Comparison of five modelling techniques to predict the spatial distribution and abundance of seabirds. Biological Conservation 156: 94–104. doi:10.1016/j.biocon.2011.11.013.

5. Worm B, Sandow M, Oschlies A, Lotze HK, Myers R a (2005) Global patterns of predator diversity in the open oceans. Science 309: 1365–1369. doi:10.1126/science.1113399.

6. Cayula JF, Cornillon P (1992) Edge detection algorithm for SST images. Journal of Atmospheric and Oceanic Technology 9: 67–80.

7. Casey KS, Evans R (2008) Global AVHRR 4 km SST for 1985-2005, Pathfinder v5.0, NODC/RSMAS. NOAA National Oceanographic Data Center.
