# Supplementary material for: Can Static Habitat Protection Encompass Critical Areas for Highly Mobile Marine Top Predators? Insights from Coastal East Africa
Source: PLoS One. 2015 Jul 17;10(7):e0133265. doi: 10.1371/journal.pone.0133265 (PMC4506016; doi:10.1371/journal.pone.0133265)
Supplement: S3 Text — (DOCX) [file pone.0133265.s009.docx]

Text S3

**Modelling results from the three ecological measurements**

Modelling occurrence data

The model with the lowest AIC (*MwlAIC*) included only 3 explanatory variables with an Akaike weight (*A_w_*) value of 0.239 in the case of GLM, indicating that a model averaging approach should be applied (Table 3). A total of 52 models were combined in the 95% confidence set and were used to estimate the average model and reduce model uncertainty. The deviance explained by MwlAIC was 9.84% and 6.28% in the train and test data respectively (Table 3). BATH100 and FRONT were the predictors with the strongest negative effect, showing the highest presence probabilities close to the 100 meter isobath and oceanic fronts (S3 Fig.). Regarding model evaluation, GLM had a good ability to discriminate between areas where dolphins were present and absent (note C-index values > 0.8; Table 3).

In the case of GAM modelling, the MwlAIC included 5 variables with an Akaike weight value of 0.264, and 13 models were averaged to reach the 95% confidence set (Table 3). REEF and FRONT were the most important variables describing dolphin occurrence; higher presence probability occurring in close proximity to the reefs and frontal systems, in shallow waters relatively close to the shelf-break (S3 Fig.). The explained deviances were higher compared to GLM with both train and test data (approximately 18% and 19%, respectively) (Table 3). Model averaged showed good model performance, but poorer than the GLM in both datasets. The weighted average (WA) consensus method values indicated that ensemble predictions were the best discriminating trained data and very similar to GLM on the cross-validation (Table 3).

Modelling sightings data

In terms of GLM output, the MwlAIC had an Akaike weight value of 0.279, and 45 models were needed to achieve the 95% confidence set towards diminishing present uncertainty (Table 3). This model explained 15% of the train data of dolphin sightings, while explaining only the 10% of the test data. BATH100 and FRONT were the most influential variables, in addition to CHLT in a minor degree, having predictive functional relationships analogous to the occurrence models (S3 Fig.). Averaged GLM models yielded good predictions for trained and tested sightings data (Table 3).

For GAM models, a similar Akaike weight value to the GLM models was obtained for the best model, but explaining a much higher percentage of the variance (27.00% in train data and 31.10% in test data) (Table 3). Sightings probability increase adjacent to the reefs, at waters depths between 5 to 30 meters which are influenced by small variations on chlorophyll among seasons (S3 Fig.). The 8 averaged models showed a high moderate performance for training and test datasets. By contrast, the ensemble predictions yielded the best model performance values for training data and comparable to GLM on the test data (Table 3).

Modelling group size data

A total of 4513 individual dolphins were counted on the 490 sightings over the four years of study period. For GLM, the MwlAIC had an Akaike weight of 0.221, and 102 models were averaged to achieve the 95% confidence set to reduce model uncertainty (Table 3). Explained deviances by the best models were comprised around 15% for both training and test data. BATH100 was the most important predictor showing the highest group size probability close to the shelf-break (S3 Fig.). Averaged group size models yielded good discrimination predictions, slightly lower compared to the GLM occurrence and sightings models (Table 3).

For GAM, the MwlAIC included the same 6 ‘non-correlated’ variables from the GAM sightings data and the addition of the SST, showing an increase of dolphin group size during seasons of low temperatures (24°-25° degrees). This explained nearly 29% of the deviance explained on training data and more than 40% for the test data (Table 3). The MwlAIC had an Akaike weight value of 0.472, and 7 models were averaged to get the 95% confidence set. These averaged models showed high moderate model performance for both datasets. Following the results from the occurrence and sightings models, the ensemble predictions improve the model performance for the training data set and get very close values to GLM on the cross-validation data (Table 3).
